# Colorectal cancer surveillance in Lynch syndrome: quality standards, emerging technology and complementary strategies

**DOI:** 10.1007/s10689-026-00608-6

**Published:** 2026-10-21

**Authors:** Joaquín Castillo-Iturra, Evelien Dekker, Francesc Balaguer

**Affiliations:** 1 Department of Gastroenterology, Hospital Clínic de Barcelona, Barcelona, Spain; 2 Institut d’Investigacions Biomèdiques August Pi i Sunyer (IDIBAPS), Barcelona, Spain; 3 Centro de Investigación Biomédica en Red de Enfermedades Hepáticas y Digestivas (CIBEREHD), Madrid, Spain; 4 Facultat de Medicina i Ciències de la Salut, Universitat de Barcelona (UB), Barcelona, Spain; 5 Department of Gastroenterology and Hepatology, Amsterdam University Medical Center, University of Amsterdam, Amsterdam, The Netherlands

**Keywords:** Lynch syndrome, Colonoscopy surveillance, Colorectal cancer, FIT, Biomarkers

## Abstract

Lynch syndrome is the most common hereditary colorectal cancer syndrome, and colonoscopy remains the cornerstone of prevention. However, surveillance is limited by missed lesions, variable procedural quality, accelerated neoplastic progression, and the continued occurrence of post-colonoscopy colorectal cancers. This review examines gene-specific differences in colorectal cancer and adenoma risk, quality standards for surveillance colonoscopy, emerging artificial intelligence technologies, and complementary non-endoscopic strategies. Contemporary studies conducted in expert settings have reported higher adenoma detection rates, reflecting improvements in colonoscopy quality. At the same time, randomized trials have shown no clear additional benefit from computer-aided detection when added to high-quality colonoscopy. Among complementary strategies, fecal immunochemical testing may support prioritization and risk stratification, although its optimal threshold, testing interval, and safety for postponing colonoscopy remain uncertain. In parallel, blood-based biomarkers for cancer prediction remain exploratory. Future progress will depend on wider implementation of quality-assured surveillance and prospective validation of adjunctive tools that can safely guide colonoscopy timing and reduce the occurrence of post-colonoscopy colorectal cancers.

## Introduction

Lynch syndrome (LS) is the most common hereditary colorectal cancer (CRC) syndrome and is caused by pathogenic variants in DNA mismatch repair (MMR) genes or by 3′ end deletions of *EPCAM* that epigenetically silence *MSH2*. Individuals with LS are at increased risk of several malignancies, although CRC remains the most frequent and the only LS-associated cancer for which an effective surveillance strategy has been established. CRC prevention in LS has traditionally relied on colonoscopic surveillance, mainly through the detection and resection of adenomas, which are considered the principal precursor lesions. Adenomas are thought to progress more rapidly in LS than in sporadic CRC [1], which shortens the window for detection at colonoscopy and provides the rationale for intensive surveillance at short intervals. In addition, aspirin reduces CRC incidence in LS, supporting chemoprevention as a complementary preventive strategy alongside high-quality colonoscopy [2]. Nevertheless, CRC still occurs in a proportion of LS carriers despite regular surveillance, underscoring the need to better understand the biology of precursor lesions and to optimize prevention strategies.

During colonoscopy, polyps are more likely to be missed when they are small, flat or non-polypoid, and located in the proximal colon, features that are particularly common in LS. This subtle morphology and distribution likely contribute to the high adenoma miss rates reported in tandem colonoscopy studies, in which two same-day colonoscopies are performed to estimate the proportion of lesions missed during the first examination. In LS, adenoma miss rates of up to 48% have been reported [3–7], compared with approximately 25% for small adenomas in average-risk populations [8].

The clinical relevance of missed lesions is further increased by their biological features: consistent with the accelerated adenoma to carcinoma pathway noted above [1], LS adenomas may display high-grade dysplasia even when small (<10 mm). Consequently, even diminutive lesions cannot be assumed to be innocuous, and LS surveillance requires both short intervals and colonoscopies performed to high-quality standards.

Despite colonoscopy surveillance, post-colonoscopy CRC remains an important clinical problem. Its occurrence is likely explained by the combined effect of missed or incompletely resected lesions, rapid adenoma-to-carcinoma progression, variability in colonoscopy quality, and, in some cases, the existence of a non-polypoid carcinogenic pathway that may be more difficult to prevent by colonoscopy [9]. Understanding these limitations requires looking beyond surveillance alone. CRC risk in LS differs substantially by gene, and recent studies also show that risk estimates may vary according to the population and methodology used to measure them. Adenoma risk follows a similar pattern, with gene-specific differences that may help explain variation in subsequent CRC risk. This review therefore starts by revisiting CRC and adenoma risk in LS, before focusing on how surveillance quality affects prevention. It then examines whether emerging technologies such as artificial intelligence and complementary strategies such as fecal immunochemical test (FIT) can add value by improving detection, prioritizing colonoscopy, or supporting more individualized surveillance pathways.

## Gene-specific heterogeneity in colorectal cancer and adenoma risk

The clinical phenotype of LS varies substantially according to the affected mismatch repair gene. CRC risk is consistently higher and age at onset younger in *MLH1* and *MSH2* carriers than in *MSH6* and *PMS2* carriers, although the magnitude of these differences varies across populations and according to the study design used to estimate penetrance. Although *EPCAM* is not itself a mismatch repair gene, 3′-end deletions can lead to epigenetic silencing of *MSH2* and confer a CRC risk broadly comparable to that observed in *MSH2* carriers. Accordingly, current surveillance recommendations generally follow the same strategy as for *MSH2*. However, specific data on adenoma detection and surveillance outcomes in *EPCAM* deletion carriers remain limited, as most studies either group them with *MSH2* carriers or do not report them separately.

The issue of gene-specific cancer risk was recently addressed in a systematic review and meta-analysis that classified available studies according to their design and main sources of bias [10]. Retrospective family studies may overestimate cancer risk because families with a strong cancer history are more likely to be identified and included. The studies were therefore further classified according to whether they corrected for ascertainment bias. Prospective cohorts, such as the Prospective Lynch Syndrome Database (PLSD), reduce this retrospective bias by following carriers after entry into surveillance, but they still include individuals identified through clinical pathways [11]. Population-based cohorts further reduce phenotype-based selection, although they may introduce other biases, such as healthy volunteer and survivor effects [12]. These different approaches and their corresponding CRC risk estimates are summarized in Table 1.

**Table 1:** Cumulative CRC risk at 70 years by gene, sex, and study design (adapted from Campoy et al.)

	Retrospective studies without ascertainment-bias correction		Retrospective studies with ascertainment-bias correction		Prospective cohort (PLSD)		Population-based cohort (UK Biobank)	
Gene	Female (%)	Male (%)	Female (%)	Male (%)	Female (%)	Male (%)	Female (%)	Male (%)
*MLH1*	50.6	60.1	33.8	42.2	42.1	52.8	38.2	53.5
*MSH2*	53.7	57.4	33	41.5	39.8	51	32.8	31
*MSH6*	22.2	43.1	10	16.8	17.3	13.5	11	14.6
*PMS2*	–	–	9	12.4	8.5	10.5	5	3.4

*PLSD* Prospective Lynch Syndrome Database

In retrospective studies with ascertainment bias correction, CRC risk estimates were generally lower than those reported in uncorrected family studies and closer to estimates from prospective or population-based designs. For example, in ascertainment-corrected retrospective family studies, CRC risks by age 70 years were estimated at approximately 34%–42% for *MLH1* carriers, 33%–42% for *MSH2* carriers, 10%–17% for *MSH6* carriers, and 9%–12% for *PMS2* carriers. In the PLSD, cumulative CRC risks by age 70 years were estimated at approximately 42%–53% for *MLH1* carriers, 40%–51% for *MSH2* carriers, and 14%–17% for *MSH6* carriers. In a population-based setting, the UK Biobank identified 830 carriers of pathogenic MMR variants among 454,756 participants and reported an overall CRC cumulative incidence of 11.8% by age 70 years, compared with 1.7% among non-carriers. The cohort was predominantly composed of *PMS2* and *MSH6* carriers, which should be considered when interpreting the overall estimate. Taken together, these designs are converging rather than competing: ascertainment-corrected retrospective estimates now approximate those from prospective and population-based cohorts, indicating that earlier studies overestimated absolute risk. What persists across all designs is a consistent gradient by gene and sex, with substantially higher CRC risk in *MLH1* and *MSH2* carriers, large enough to justify gene-differentiated surveillance rather than a uniform protocol.

Age at CRC onset may also be influenced by birth cohort effects. This question is important because earlier CRC diagnosis in successive generations can be interpreted as genetic anticipation, whereas it may instead reflect broader population-level changes in CRC incidence. A recent study evaluated this distinction in 240 individuals with LS from two academic registries. Later birth year was associated with CRC diagnosis at a younger age (HR 1.09; 95% CI 1.05–1.12). In an expanded pedigree-based analysis including 4645 family members, birth year remained associated with earlier CRC onset, whereas family generation was not [13]. These findings support a birth cohort effect rather than true genetic anticipation, although larger validation studies are needed before this can be translated into changes in surveillance initiation.

Gene-specific differences have also been reported in adenoma and advanced adenoma detection. An earlier multicenter cohort of 2747 LS carriers reported a lower 10-year cumulative incidence of adenomas and advanced adenomas in *MLH1* compared with *MSH2* carriers (32.2% vs. 44.2% and 7.7% vs. 17.8%, respectively) [14]. More recently, a multicenter study including 1072 LS carriers without prior CRC and 4357 colonoscopies found no significant differences in adenoma or advanced adenoma detection between *MLH1* and *MSH2* carriers, although *MLH1*/*MSH2* carriers overall had a higher advanced adenoma detection rate than *MSH6*/*PMS2* carriers (14.5% vs. 11.9%; *p* = 0.04), as well as a higher 10-year cumulative advanced adenoma detection rate (21.6% vs. 19.7%; *p* = 0.04) [15]. Several differences between the study populations may partly explain these apparently discordant findings. In the Engel et al. cohort, 38% of participants had a previous CRC and many had therefore undergone colorectal resection, reducing the amount of colon at risk for subsequent adenoma development and detection. In addition, *MLH1* carriers were heavily represented by Finnish founder-variant families, and detailed colonoscopy quality indicators were not available. These factors may have influenced adenoma detection and complicate direct comparison between studies.

The lower average risk for colorectal neoplasia observed in *MSH6* and *PMS2* carriers does not exclude clinically relevant neoplasia at young ages. This issue has gained importance as several guidelines have moved toward later initiation of colonoscopy for *MSH6* and *PMS2* carriers. In a retrospective multicenter study of 285 *MSH6* or *PMS2* carriers who underwent colonoscopy before age 50, neoplastic lesions were detected in 24% of 824 procedures, including CRC in 4.1%. Among 122 procedures performed before age 30, neoplasia was detected in 13%, including CRC in 3.3% and advanced adenomas in 2.4%. All CRCs detected before age 30 occurred before LS diagnosis [16]. Although the selected tertiary-center population limits generalizability, these findings show that clinically relevant neoplasia, including CRC, may occur before the currently recommended age for surveillance initiation in some carriers of lower-penetrance genes. Importantly, all CRCs diagnosed before age 30 occurred before LS diagnosis and therefore outside an established LS surveillance program. While these data cannot demonstrate that earlier colonoscopy would have prevented these cancers, they provide information on the natural history of *MSH6*/*PMS2*-associated syndrome and raise the question of whether current recommendations regarding the age at initiation of surveillance are appropriate for all carriers.

Collectively, these data show that colorectal neoplasia risk in LS is heterogeneous and cannot be explained by the affected MMR gene alone. Although gene and sex are major determinants of CRC and advanced adenoma risk, other factors—including age, birth cohort, personal history of CRC or advanced adenomas, repeated detection of advanced neoplasia, family history, surgical history, adherence to surveillance, aspirin use, lifestyle factors, and the quality of previous colonoscopies—may further modify individual risk. Family history may provide additional context for individual risk assessment, although its independent contribution to CRC risk beyond the affected MMR gene remains insufficiently established [17]. Importantly, neither family history nor these other potential risk modifiers has yet been validated as a basis for changing the age at initiation or the interval of colonoscopy surveillance. Future personalized strategies will therefore require prospective validation of combinations of inherited and dynamic clinical risk factors rather than reliance on any single variable.

## Quality standards

The effectiveness of colonoscopy surveillance in LS depends not only on repeated examinations, but also on the consistent delivery of high-quality procedures. Colonoscopy quality is a multidimensional concept that includes adequate bowel preparation, complete examination with cecal intubation, thorough mucosal inspection with sufficient withdrawal time to carefully look behind folds and in the flexures, optimal lesion detection, complete and safe resection of detected lesions, and adherence to recommended surveillance intervals [18]. These factors are particularly relevant in LS, where precursor lesions may be subtle, flat, and rapidly progressive, so that missed or incompletely resected lesions are more likely to result in post-colonoscopy CRC. Therefore, quality standards should be considered an integral component of LS surveillance rather than a procedural detail.

Available data indicate that quality standards derived from colonoscopy in general are not consistently achieved in LS surveillance. In a retrospective multicenter study including 893 individuals and 4177 colonoscopies, only 40.4% of carriers met minimum surveillance quality criteria when these were defined as complete examination, adequate bowel preparation, and all surveillance intervals shorter than 3 years; this proportion decreased to 28.2% when a stricter interval of less than 2 years was required [19]. More recently, a UK cohort at a tertiary center reported excellent technical performance, with cecal intubation and adequate bowel preparation achieved in 97.6% and 96.3% of procedures, respectively. However, only 70.4% of colonoscopies were performed within 2.25 years of the previous examination [20]. These findings highlight that high-quality LS surveillance requires both technically optimal colonoscopy and reliable adherence to recommended surveillance intervals. Even when procedural quality is high, delays between examinations may compromise the preventive potential of surveillance. Such interval delays may be patient-related or service-related, a distinction that is also relevant when evaluating non-endoscopic strategies intended to reduce surveillance burden.

Among colonoscopy quality indicators in the general population, the adenoma detection rate (ADR) is the most extensively validated. In average-risk screening, ADR is inversely associated with the risk of post-colonoscopy CRC and CRC-related mortality [21]. However, the use of ADR as a quality benchmark in LS surveillance is less straightforward. LS carriers usually enter surveillance at younger ages and undergo repeated colonoscopies at short intervals, commonly every 1–3 years across guidelines [22–25]. Because LS carriers do not appear to develop more adenomas than average-risk individuals, and repeated short-interval colonoscopy progressively removes prevalent lesions, per-procedure ADRs during LS surveillance may be lower than those observed at first-time average-risk screening, even when mucosal inspection is meticulous. This pattern was confirmed in the first systematic review and meta-analysis of neoplasia detection in LS, which included 45,421 colonoscopies in 15,227 carriers and reported a pooled ADR of 23.3% (95% CI 20.6–26.3). ADR was higher in high-quality procedures (26.3%) and in clinical trials conducted under standardized quality conditions (30.7%) than in retrospective observational cohorts (18.5%) [26]. Interpretation is further complicated because ADRs reported in LS studies often reflect adenoma detection per procedure, per surveillance round, or per study cohort, rather than endoscopist-level performance. Consequently, ADR thresholds derived from average-risk screening cannot be applied directly to LS surveillance, and no validated LS-specific benchmark has been established. Endoscopist competence may still be captured by ADR achieved in average-risk or screening colonoscopy, whereas quality assessment in LS may additionally require dedicated indicators that account for the distinctive age distribution, surveillance intensity, lesion phenotype, and cancer risk of this population.

Beyond ADR, several other quality indicators appear to influence the effectiveness of LS surveillance. In the multicenter study described above, adequate bowel preparation, complete examination with cecal intubation, and the use of chromoendoscopy were independently associated with higher adenoma detection. However, this finding has not been consistently confirmed in randomized studies. An individual-patient-data meta-analysis found no clear advantage of dye-based chromoendoscopy over white-light endoscopy for adenoma detection [27]. Evidence for virtual chromoendoscopy is more limited but potentially more encouraging: in a randomized trial, linked-color imaging increased the detection of proximal and ≤5 mm adenomas compared with high-definition white-light endoscopy (HD-WLE) [28]. Overall, current evidence does not support routine dye-based chromoendoscopy in LS surveillance, while the potential added value of virtual chromoendoscopy requires further confirmation. Importantly, surveillance intervals shorter than 3 years were associated with a significantly lower risk of post-colonoscopy CRC (OR 0.35; 95% CI 0.14–0.97) [19]. These findings suggest that CRC prevention in LS depends on a combination of lesion detection, complete mucosal assessment, and timely repetition of examinations, rather than on any single quality metric.

Quality-assured surveillance therefore requires not only high-quality colonoscopy at the procedural level, but also organized care pathways that ensure adherence to recommended intervals and continuous monitoring of outcomes. The integration of LS surveillance into England’s national bowel cancer screening infrastructure illustrates this model, combining accredited endoscopists, standardized referral and recall systems, dedicated training, audit, and systematic monitoring of colonoscopy quality and surveillance outcomes [29]. Such structured programs may be particularly relevant in LS, where small lapses in procedure quality or delays in surveillance can have disproportionate consequences because of the rapid progression potential of precursor lesions.

## Emerging technologies

Artificial intelligence (AI), particularly computer-aided detection (CADe), has become an important technological development in colonoscopy. CADe systems identify and flag suspected lesions during real-time examination, whereas computer-aided diagnosis (CADx) systems assist in the optical characterization of detected lesions. In the average-risk population, CADe has consistently increased adenoma detection. A systematic review and meta-analysis including 21 randomized trials and 18,232 average-risk patients showed that CADe increased ADR from 35.9 to 44.0% and reduced adenoma miss rate by approximately 55%. However, this benefit was mainly driven by increased detection of small adenomas, without a clear increase in advanced adenoma detection, and was accompanied by a higher number of non-neoplastic polypectomies [30]. Importantly, most of this evidence derives from controlled randomized trials, and comparable improvements have not been consistently reproduced when CADe has been implemented in routine clinical practice, suggesting that its real-world effectiveness may depend on baseline endoscopist performance, adherence to the technology, workflow integration, and local quality standards.

These findings generated particular interest in LS, where CADe could theoretically be useful because adenomas are often small, flat, and difficult to detect, while even diminutive lesions may have advanced histological features. The first randomized pilot trial in LS, the CADLY study, compared real-time CADe-assisted colonoscopy with HD-WLE. Although ADR was numerically higher with CADe, the difference was not statistically significant (36.0% vs. 26.1%; *p* = 0.379). CADe nevertheless detected a significantly higher proportion of flat adenomas, providing an initial signal that it might be particularly useful for subtle lesions commonly encountered in LS [31].

However, in the larger TIMELY trial, a multicenter randomized controlled trial including 430 individuals with LS, CADe-assisted colonoscopy using Medtronic GI Genius™ did not improve the mean number of adenomas detected per colonoscopy compared with HD-WLE alone. The mean number of adenomas per colonoscopy was identical in both groups (0.64 vs. 0.64; *p* = 0.87). ADR was also not significantly different between CADe-assisted colonoscopy and HD-WLE (33% vs. 36%; *p* = 0.42) [32]. Notably, ADR in the HD-WLE group was among the highest reported in LS surveillance.

More recently, the CADLY2 multicenter randomized controlled trial evaluated CAD EYE (Fujifilm, Tokyo, Japan) in 733 individuals with LS [33]. Consistent with the TIMELY trial, CADe did not significantly improve ADR compared with HD-WLE (33.8% vs. 30.9%; *p* = 0.409). Taken together, current evidence does not support routine CADe-assisted colonoscopy in LS surveillance, as its incremental benefit appears limited when procedures are performed by experienced endoscopists with rigorous mucosal inspection. The consistently high ADRs in the control groups of contemporary LS trials reinforce the importance of operator expertise and procedural quality.

Beyond detection, CADLY2 also evaluated CADx for optical differentiation between neoplastic and non-neoplastic lesions. Although CAD EYE showed good diagnostic performance, with a sensitivity of 84.4% (95% CI 80.6–87.6) and specificity of 90.9% (95% CI 88.9–92.6), endoscopist optical diagnosis achieved higher sensitivity with comparable specificity. These findings suggest that CADx may provide limited incremental value over expert optical diagnosis. Nevertheless, the role of AI will probably continue to evolve as detection and characterization systems improve, with potential value in reducing inter-endoscopist variability and improving CADx capabilities.

## Complementary strategies

A range of non-endoscopic approaches has been investigated as a complement to colonoscopy, with the aim of reducing the burden of repeated lifelong examinations. A systematic review of non-endoscopic surveillance found radiological modalities (CT and MR colonography) unsuitable, owing to poor sensitivity for smaller lesions, whereas biomarker-based strategies, including FIT, plasma methylated *SEPTIN9*, fecal tumor DNA markers and fecal microbiome signatures, appeared promising but remained understudied [34]. Such approaches may serve two distinct purposes: the detection of prevalent neoplasia to guide the timing of colonoscopy, exemplified by FIT, and the prediction of future cancer incidence to individualize surveillance.

### Fecal immunochemical test

Among these biomarker-based strategies, FIT is attractive in LS because it is non-invasive, inexpensive, scalable, and already integrated into population-based CRC screening programs [35]. FIT is not intended to replace scheduled colonoscopy but may help refine surveillance pathways. It could identify individuals who may benefit from earlier colonoscopy and those whose low short-term risk may support evaluation of colonoscopy postponement. This is particularly relevant because LS carriers may undergo 20–30 colonoscopies during their lifetime, while effective surveillance also depends on timely access, adherence, and patient acceptability.

The first real-world evaluation of FIT in LS came from an emergency service protocol implemented in England during the COVID-19 pandemic, when endoscopy capacity was severely restricted. In this setting, FIT was used to prioritize colonoscopy among LS carriers. Individuals with fecal hemoglobin (f-Hb) <10 µg Hb/g feces were scheduled for colonoscopy within 6–12 weeks, whereas those with f-Hb ≥10 µg/g were triaged through an urgent suspected cancer pathway, with colonoscopy scheduled within 30 days. Among 339 eligible participants, 60 (17.7%) had f-Hb ≥10 µg/g, and this group underwent colonoscopy substantially earlier than FIT-negative individuals, with a median time of 49 versus 122 days. Advanced neoplasia was observed more frequently among FIT-positive than FIT-negative individuals (14.0% vs. 8.1%), and CRC was detected in 4 FIT-positive and 3 FIT-negative participants. Importantly, the three CRCs detected in FIT-negative individuals were diagnosed after substantial delays beyond the protocol window, with a mean time to colonoscopy of 294 days, and were small lesions (<15 mm). Consequently, these cases cannot be used to directly estimate FIT sensitivity within the intended triage pathway. Overall, this experience suggested that FIT may be useful as a triage tool when colonoscopy resources are limited, supporting further evaluation of FIT as a complementary strategy to colonoscopy surveillance [36].

More robust diagnostic data have been provided by a prospective multicenter study in which 217 individuals with LS underwent FIT before high-quality surveillance colonoscopy. At thresholds of 2.5 and 4.1 µg Hb/g feces, FIT detected all four CRCs and four of five advanced adenomas, corresponding to a sensitivity of 89% and a negative predictive value of 99% for the combined endpoint of CRC and advanced adenomas. At 4.1 µg/g, specificity reached 94%. However, only one of four advanced serrated lesions was detected. At this threshold, modeling suggested that two advanced neoplastic lesions per 100 individuals tested would be missed [37].

Acceptability data also support the feasibility of FIT-based strategies. In a mixed-methods evaluation of individuals with LS, 85% of participants rated FIT positively and 90% were confident that they could use the test correctly. Participants valued its convenience and perceived it as a potential means of reducing delays or providing reassurance between colonoscopies [38].

FIT remains an investigational tool in LS surveillance. Available evidence suggests potential utility for prioritization and risk stratification when colonoscopy access is limited or surveillance is delayed. Beyond this triage role, the potential value of FIT as a complement to scheduled colonoscopy is being prospectively evaluated in the FIT for Lynch study, a single-arm, multicenter study assessing annual FIT alongside biennial colonoscopy in LS carriers [39]. A second prospective multicenter study, currently recruiting in the United States (NCT06898996), is evaluating the accuracy of FIT relative to surveillance colonoscopy in LS carriers. Before FIT can be incorporated into routine surveillance algorithms, further validation is needed to establish the optimal f-Hb cut-off and to define its role and safety.

### Frameshift mutations in circulating cell-free DNA

Blood-based biomarkers are also being explored as complementary tools. The rationale for this approach derives from the biology of mismatch repair deficiency, which leads to microsatellite instability (MSI) and the accumulation of recurrent frameshift mutations (FSMs) at coding mononucleotide repeats. These recurrent alterations are of growing interest in LS as molecular consequences of MMR deficiency and as a source of shared frameshift peptides with immunogenic potential [40, 41]. In this context, one proposed approach is the detection of recurrent FSMs in circulating cell-free DNA (cfDNA). In a proof-of-concept study involving a heterogeneous group of MSI-high/MMR-deficient cases, a 122-target FSM panel detected recurrent FSMs in cfDNA, including in one asymptomatic LS carrier, while showing minimal background detection in healthy controls. Although the assay distinguished MSI-high/MMR-deficient cases from healthy controls with an AUC of 0.94, its performance and biological meaning in asymptomatic carriers remain uncertain [42]. Larger longitudinal studies are required to determine whether circulating FSMs can provide clinically useful information for surveillance.

### Circulating microRNAs and multi-omic biomarkers

Circulating microRNAs have also been explored. These short, noncoding RNA molecules regulate gene expression and have been widely investigated as cancer biomarkers in sporadic settings [43]. In a Finnish pilot study, a circulating microRNA signature was evaluated in 110 cancer-free LS carriers followed over a 4-year prospective surveillance period, during which 18 developed cancer (9 CRC). The authors derived a five-microRNA signature whose risk-sum score was associated with cancer incidence (HR 2.72; 95% CI 1.64–4.52; C-index 0.72) [44]. The same group subsequently applied a bi-omic approach integrating circulating microRNAs and metabolites in 116 cancer-free LS carriers (45 with a previous cancer history), of whom 17 developed cancer (8 CRC) over a 5.8-year surveillance period. Three biomarkers—two microRNAs (hsa-miR-101-3p and hsa-miR-183-5p) and triglycerides in HDL particles—independently predicted cancer, with elevated levels indicating higher risk (C-index 0.76) [45]. Unlike FIT, which detects prevalent neoplasia, these biomarkers are intended to predict future cancer occurrence and could in principle inform the intensity and timing of surveillance. However, these approaches remain exploratory and require validation before any surveillance application can be considered.

## Future directions

**Fig. 1:**
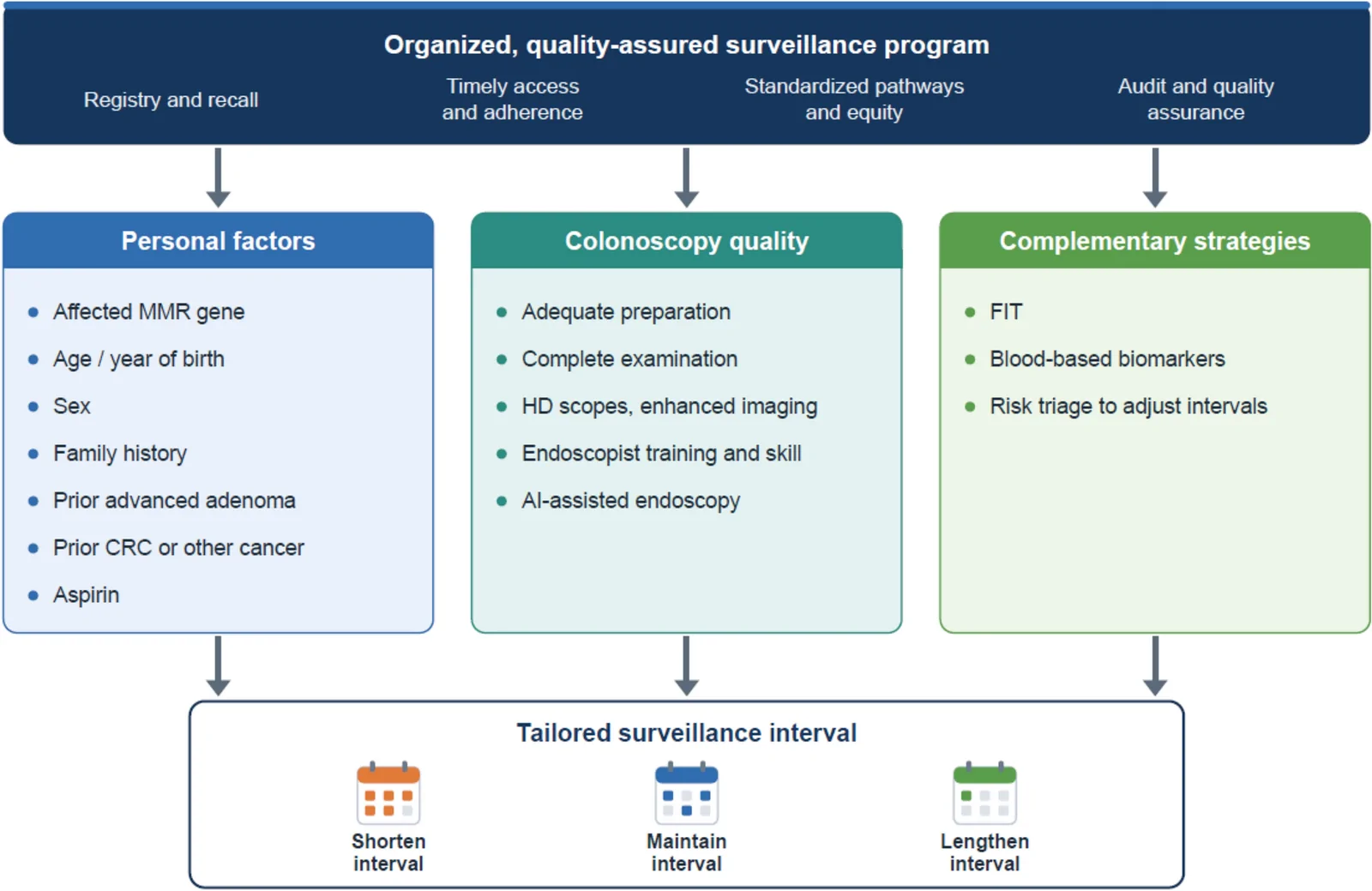
Future directions in CRC surveillance in LS: a possible model for individualized care. MMR, mismatch repair; CRC, colorectal cancer; HD, high-definition; AI, artificial intelligence; FIT, fecal immunochemical test

Substantial progress has been made in LS surveillance, as reflected by the higher per-procedure ADRs reported in contemporary studies. Further improvement will depend on the wider implementation of quality-assured surveillance programs and on the prospective validation of adjunctive tools that can safely guide colonoscopy timing. At present, neither AI-assisted colonoscopy has demonstrated a clear incremental benefit over high-quality examination, nor has any non-endoscopic test—FIT, blood-based biomarkers or fecal microbiome signatures—established a safe role in modifying surveillance intervals. For liquid biopsy approaches in particular, this evidence is still awaited: the forthcoming PREDI-LYNCH trial (NCT07360834) will prospectively evaluate whether circulating biomarkers can safely reduce the burden of invasive surveillance in LS carriers. Because prospective evaluation of modified surveillance intervals requires large cohorts and prolonged follow-up, modeling studies may help define how such tools are used: a recent decision-analytic model in *PMS2* carriers found that triennial colonoscopy from age 30, alone or with interim FIT, substantially reduced lifetime colonoscopies and costs with minimal change in CRC incidence or mortality [46]. Future studies should establish whether these approaches genuinely improve lesion detection or risk stratification and ultimately reduce post-colonoscopy CRC and should quantify the impact of structured quality-improvement programs, for which England’s national model may provide valuable real-world evidence. Regardless of how detection and risk stratification evolve, consistent adherence to the recommended—and increasingly personalized—surveillance schedule remains the single most important determinant of effectiveness; achieving it will require organized, quality-assured programs with reliable recall systems, minimization of both patient- and service-related delays, and clear communication of individualized intervals to carriers and clinicians. Taken together, these elements outline a possible framework in which an organized surveillance program, gene- and individual-level risk, colonoscopy quality, and complementary strategies could converge into a tailored surveillance approach (Fig. 1).

## Data Availability

No datasets were generated or analysed during the current study.
